# First-Year Law Students’ and Teacher’s Questioning in Class

**DOI:** 10.3389/fpsyg.2019.00611

**Published:** 2019-03-21

**Authors:** Luísa Ribeiro, Pedro Rosário, Iněs Moreira, Rosário Serrão Cunha

**Affiliations:** ^1^Centro de Investigação para o Desenvolvimento Humano, Faculdade de Educação e Psicologia, Universidade Católica Portuguesa, Porto, Portugal; ^2^Escola de Psicologia, Universidade do Minho, Braga, Portugal

**Keywords:** first-year students, teacher questioning, student questioning, questioning practices, questioning perceptions

## Abstract

Classroom questioning can be considered a key factor in the promotion of student engagement. This case study explored classroom questioning practices and perceptions of a group of 47 first-year law students and their teacher. Eight lessons of 90 min were observed and audio-recorded and afterward the students and the teacher answered a questionnaire. The teacher was also interviewed. Researchers examined the number and type of questions asked by the teacher and by the students in the classroom and analyzed the students’ and the teacher’s perceptions about the importance of classroom questioning. Results indicated that the teacher and most students consider questioning important or very important for student learning. The number of questions posed by students as opposed to by their teacher was not balanced, as the teacher was responsible for 93% of the questions. The analysis of the type of questions posed by the teacher and by the students showed a predominance of low-order questions. Therefore, classroom questioning in this case study did not seem to promote students’ autonomous thinking. The current study suggests the importance of examining the teacher and students’ patterns of questioning together, analyzing its similarities and discrepancies.

## Introduction

Colleges and universities are challenged to adopt teaching and learning methods that promote students’ agency and autonomy, as well as high levels of thinking skills and student engagement (Pedrosa-de-Jesus et al., 2012). Classroom questioning can be identified as a key element of the response to this demand, transforming students into active learners rather than passive receivers of information (Van Drie and Van Boxtel, 2008; Haydon and Hunter, 2011; Song et al., 2017). Questioning represents a noteworthy part of teaching and teacher-student interaction (Ernst-Slavit and Pratt, 2017) and has a significant impact on students’ cognitive engagement, depending on questioning patterns (Noor et al., 2012; Smart and Marshall, 2013). In fact, questioning is considered an essential tool for effective teaching in higher education (Maphosa and Wadesango, 2016).

Research shows the relevance of questioning as a teaching technique that contributes to engaged learning and the promotion of deeper-level thinking by students (Erdogan and Campbell, 2008; Rosário et al., 2010; Tofade et al., 2013). Questioning can improve students’ critical thinking and problem solving skills, both of which are fundamental competencies for success in learning and in life. Educators are challenged to contribute to students’ own questioning and problem solving abilities (Wechsler et al., 2018) and questioning technique is a very helpful classroom practice throughout the education system. Moreover, questioning can be used in the classroom in order to assess students’ learning. For example, Singh et al. (2017) found that oral questioning is the most used method of assessment by teachers. However, the potential positive effects of questioning depend on the way this strategy is used by the teacher (Maphosa and Wadesango, 2016).

In different levels of schooling, several studies emphasize the lack of high-order questions and the predominance of factual or recall-type questions, as well as procedural questions (Albergaria-Almeida, 2010; Pedrosa-de-Jesus et al., 2012). The stimulation of higher cognitive processes and critical reasoning skills is an important educational issue. This conveys implications for teachers’ professional development (Maphosa and Wadesango, 2016). Recently, a study on acquisition of soft skills by teachers as a way to improve their quality of teaching, and consequently students’ achievement, showed that communication skills are the most important to acquire, according to teachers’ perspective (Tang, 2018). As Van Drie and Van Boxtel (2008) stated, “Not all questions ask for the transformation of knowledge and information.” (p. 91). Literature showed that different levels and types of questions (Logtenberg et al., 2011) can lead to different questioning patterns in the classroom and thus lead to different outcomes in the teaching and learning process (Noor et al., 2012).

### Purpose of the Study

Literature has highlighted the importance of teacher and student questioning for both teaching and learning (Ong et al., 2016), yet the majority of the studies are focused on teachers’ questioning, rather than on student-generated questions (Whittaker, 2012). Furthermore, the combined analysis of questioning patterns of both the participants in the teaching and learning process has received limited attention, especially in college. The current study expands upon existing literature by simultaneously examining the use of questioning by the teacher and their students. It also examines questioning practices and perceptions, enabling the joint analysis of teacher and students questions in the classroom and their perceptions about the importance of questioning. This research analyses the ways a university teacher and their first-year undergraduate law students use questions during lectures and practical lessons, and thus responds to the need for further research about domain-specific questioning processes (Logtenberg, 2012).

## Materials and Methods

### Participants

Participants were 47 first-year law students (48.9% women, aged between 17 and 26 years, *M* = 19.50, *SD* = 2.15) and their respective teacher (male, 24 years of teaching experience) in a Portuguese private urban university that offers different study areas and degree levels. Among these students, 63.8% were attending college for the first time.

### Instruments and Procedures

The case study was developed in a specific core subject of law course. Four 90-min lectures and four 90-min practical lessons, during first semester, were observed and audio-recorded. All the questions posed by the students and by the lecturer and their respective answers were transcribed.

In the last class, all participants indicated the perceived value they attribute to teacher questioning (one item) and student questioning (one item) in the classroom. The scale for the two items contained five possible answers ranging from 1, meaning *not at all important*, to 5, meaning *very important*.

After the observational and questionnaire data collection, a semi-structured interview was conducted with the teacher to give him feedback about the findings and explore possible explanations to the results. Interview questions referred to the importance of teacher’s and students’ questioning in the classroom and its main functions. The teacher presented his explanations for the observed data and reflected on the kind of questions he used to pose to his students.

All the elements of this case study contributed to the purpose of the study. Besides the analysis of the typology of the questions posed by the teachers and by the students, the questionnaire allowed researchers to analyze participants’ perceptions about the value they attributed to teacher’s and students’ questioning, and the interview enabled them to collect relevant data about teacher perceptions of classroom questioning, reflecting from data from his own classes.

We carried out this study following the recommendations from the ethics committee of the University of Minho. Participants gave written informed consent to participate in the research in accordance with the Declaration of Helsinki. Participants were informed about the objectives of the investigation and confidentiality and anonymity were assured. In addition, participation in our study was voluntary and researchers informed students about data usage.

### Data Analysis

The number and the type of questions asked both by the teacher and by the students were analyzed. All issues recorded, after analysis, were categorized. These categories were created according to a semi-inductive process, keeping in mind the typologies consulted in the literature review (e.g., Harrop and Swinson, 2003; Erdogan and Campbell, 2008; Eshach et al., 2014). The selected categories were relevant to the observed data in this case study. All question phrases were considered, even those that were not real questions, and each question was included in only one category. One researcher collected all of the observational data. The categorization was discussed with two additional researchers until total interobserver agreement.

The perceptions of the teacher and the students about the importance given to classroom questioning were analyzed (descriptive statistics) alongside the qualitative data collected through the teacher interview (content analysis).

## Results and Discussion

The teacher’s and the students’ questions were classified in three categories (see Table 1). Questions were coded as *pseudo questions* when there was no expectation of a response and no waiting time was given after the question. *Procedural questions* referred to course functioning. *Academic* questions referred to the learning material and intended to support students’ learning and to build or assess students’ knowledge.

**Table 1:** Types of questions asked by the teacher and the students.

Question type		Sample question (teacher)	Sample question (students)
*Pseudo* questions		… Right?/Isn’t it?	
Procedural questions	Monitoring students’ attendance	Is Mary in the class?	
	Managing students’ behavior	A deck of cards, perhaps?	
	Clarifying students’ assessment conditions	Are you participating in continuous assessment?	Could it appear in the exam a question about this topic?
Academic questions	Identifying students’ doubts	Is there any doubt?	
	Checking clarity of teacher’s exposition	Understood?	
	Emphasizing ideas	What does the norm say to us? The norm says that...	
	Reacting to a student’s statement	Do they help to interpret the norm?	
	Content related closed questions	Is it a universal or a local norm?	But the fine is always in money?
	Content related open questions	In this case, what does it seem to you?	Could you please further explain the difference between judicial activity and administrative activity?
	Relating contents	Have you already learned this in another subject?	
	Assessing students’ knowledge	John, what are prohibitive norms?	


Considering the eight lessons observed (see Table 2), the teacher asked 913 questions, of which 456 were *pseudo* questions (50.0%), 356 academic questions (39.0%), and 101 procedural questions (11.1%). The average number of teacher questions per class was 57.1 (excluding *pseudo* questions).

**Table 2:** Number and type of questions asked by the teacher and the students, which emerged from eight classroom observations (four lectures and four practical lessons).

		Teacher’s questions						Students’ questions					
		Total lectures	*M(SD)* lectures	Total practical lessons	*M(SD)* practical lessons	Total teacher	*M(SD)* teacher	Total lectures	*M(SD)* lectures	Total practical lessons	*M(SD)* practical lessons	Total students	*M(SD)* students
*Pseudo* questions		278	69.5 (13.1)	178	44.5 (7.2)	456	57.0 (16.6)						
Proceduralquestions	Monitoring students’ attendance			94	23.5 (9.1)	94	11.8 (13.9)						
	Managing students’ behavior	1	0.3 (0.5)			1	0.1 (0.4)						
	Clarifying students’ assessment conditions			6	1.5 (1.7)	6	0.8 (1.4)	3	0.8 (1.5)	1	0.3 (0.5)	4	0.5 (1.1)
Academicquestions	Identifying students’ doubts	29	7.3 (3.6)	12	3.0 (0.8)	41	5.1 (3.3)						
	Checking clarity of teacher’s exposition	70	17.5 (6.6)	22	5.5 (1.7)	92	11.5 (7.8)						
	Emphasizing ideas	74	18.5 (7.9)	40	10.0 (4.6)	114	14.3 (7.5)						
	Reacting to a student’s statement			1	0.3 (0.5)	1	0.1 (0.4)						
	Content related closed questions			7	1.8 (3.5)	7	0.9 (2.5)	16	4.0 (3.6)	9	2.3 (2.6)	25	3.1 (3.0)
	Content related open questions			6	1.5 (3.0)	6	0.8 (2.1)	22	5.5 (3.7)	18	4.5 (3.7)	40	5.0 (3.5)
	Relating contents	8	2.0 (1.6)			8	1.0 (1.5)						
	Assessing students’ knowledge			87	21.8 (8.5)	87	10.9 (12.9)						
Total of procedural questions		1	0.3 (0.5)	100	25.0 (10.4)	101	12.6 (14.9)	3	0.8 (1.5)	1	0.3 (0.5)	4	0.5 (1.1)
Total of academic questions		181	45.3 (11.3)	175	43.8 (13.2)	356	44.5 (11.4)	38	9.5 (6.1)	27	6.8 (5.5)	65	8.1 (5.6)
Total of questions (excluding *pseudo* questions)		182	45.5 (10.8)	275	68.8 (15.5)	457	57.1 (17.6)	41	10.3 (7.0)	28	7.0 (5.6)	69	8.6 (6.1)


*The zero values were omitted, facilitating the reading of the table*.

In lectures, the *pseudo* questions category was the most prevalent (63.4%), followed by the emphasizing questions (16.1%), questions designed to check clarity of the exposition (15.2%), questions identifying students’ doubts (6.3%), and questions relating contents (0.2%). Content related questions, either open or closed, with the objective of constructing knowledge and helping students to think autonomously, were not observed. The teacher’s lectures could be characterized as utilizing an expository method, in which questions were mainly used to emphasize the discourse, as the teacher frequently answered his own questions (Tofade et al., 2013). This transmissive approach was often complemented with an effort to confirm that students were following and understanding the contents being taught. Therefore, the interaction between the teacher and the students was mainly focused on facilitating the presentation of the learning contents and checking for students’ understanding.

Throughout the practical classes, *pseudo* questions were the most utilized category of questions (39.3%). The next most common categories were monitoring students’ attendance questions (20.8%), questions assessing individual students’ knowledge (19.2%), emphasizing questions (8.8%), questions checking exposition clarity (4.5%), questions identifying students’ doubts (2.6%), and finally content related questions: closed questions (1.5%) and open questions (1.3%).

In practical lessons, the teacher opted for assessing students’ knowledge orally, asking questions about the learning material. Researchers observed an intense interaction between teacher and students, as students were individually questioned, in the context of a continuous assessment modality (Chin, 2006; Singh et al., 2017). In spite of this educational interaction, the questioning pattern also revealed the predominance of emphasizing questions.

In comparison to lectures, in practical lessons, content related questions, while they only represented a small percentage of the amount of total questions observed, were a novel addition. Moreover, all the content related questions corresponded to low-order questions, namely remembering and understanding, the first two categories of Bloom’s revised taxonomy (Anderson et al., 2001). Thus, in this case, like in other studies (e.g., Tofade et al., 2013), the teacher did not seem to use classroom questioning to build new knowledge and to promote students’ autonomous thinking. Instead, questioning was used primarily to assess students’ knowledge. This pattern, observed in practical lessons, illustrates the traditional IRF/E cycle, discussed by Chin (2006): teacher initiation, student response, teacher feedback or evaluation. It is worth noting that in lectures this cycle was not observed, as this kind of interaction was non-existent.

In the eight lessons observed, students asked 69 questions, namely academic questions (94.2%), and procedural questions (5.8%). The average number of students’ questions per class was 8.6.

In both lectures and practical lessons, the content related open questions were predominant (54 and 64%, respectively), followed by content related closed questions (39 and 32%), and by questions clarifying students’ assessment conditions (7 and 4%). The content related questions were mostly requests for clarification, concept definitions or repetition of certain concepts. Also in this case, questions posed by the students were low-order questions, namely Bloom’s taxonomy remembering and understanding categories (Anderson et al., 2001).

The findings also revealed that the number of questions asked by students in lectures and in practical lessons was higher before the exam and decreased after the exam, showing that students’ participation grows when they study more intensively. These data reinforce the importance of continuous assessment practices that promote regular academic engagement.

Data from the questionnaire showed that the teacher and the students agreed that classroom questioning is important or very important for student learning and that both attribute more importance to their own questions.

Through examination of the total number of questions asked in the eight classes, it was concluded that the number of questions asked by the teacher and the students was unbalanced: the teacher was responsible for 86.9% of the questions (excluding *pseudo* questions). These findings are congruent with prior studies conducted in this area (e.g., Albergaria-Almeida, 2010) pointing to the paucity of the students’ questions and to the centrality of the teacher in the teaching and learning process in the first year of university.

The analysis of the type of questions posed by the teacher and by the students revealed important similarities: the predominance of low-order questions, eliciting remembering and understanding processes; and the focus on basic comprehension of the concepts. In both cases, classroom questioning did not seem to be used in order to promote students’ autonomous thinking. These findings are consistent with literature (e.g., Tofade et al., 2013).

In the interview, teacher showed that his central concern was to transmit the contents in a very well organized and clear manner to the students, mainly because his students were first-years and were learning new concepts for the first time. This concern is consistent with the type of questions posed by the students. As referred by Logtenberg et al. (2011), lower-order questions can function as a first step to more complex questions. At this stage, asking complex questions to the students was perceived as a risky option, because of the lack of theoretical background of the students. This option can be legitimated by the literature (Smart and Marshall, 2013), but it would be important to progressively make more complex the cognitive level of the questions, as students progress in their learning. Although the participant teacher had more than 20 years of experience teaching at university, it was the first time that he had the opportunity to pause and reflect about questioning functions, types of questions, and cognitive processes elicited by questioning. The moment of the interview thus represented an opportunity for this teacher to become more reflective of his own practice.

## Conclusion

Overall the current study suggests the importance of examining the teacher’s and the students’ patterns of questioning and discussing their similarities and discrepancies. The findings of this case study suggest a mirror effect between the patterns of these two actors in the classroom. It could be important to explore the role of the modeling exerted by the teachers on their students.

The findings of the current study, while limited, suggest interesting educational implications. There is a need for learning experiences aiming at promoting metacognition processes in college classes, allowing teachers and students to explore their perceptions about teaching and learning conceptions and approaches, student classroom participation and questioning beliefs, functions, and practices (Pham and Hamid, 2013; Farrell and Mom, 2015). Higher education institutions will benefit from efforts made by teachers and students to promote complex cognitive processes in the construction of knowledge through high quality level questioning in the classroom (Smart and Marshall, 2013). Teachers are expected to have a clear focus on the cognitive processes they want to elicit in their students (Chin, 2006) and students should be trained and encouraged to ask their own questions (Logtenberg, 2012; Frambach et al., 2014). Thus, more research is needed on how to promote teacher and student question-asking skills.

## Data Availability

The datasets for this study will not be made publicly available because the researchers reported in the informed consent that the data would only be used by the research team who conducted the study.

## Ethics Statement

This study was reviewed and approved by the Ethics Committee of the University of Minho. All research participants provided written informed consent in accordance with the Declaration of Helsinki.

## Author Contributions

LR and PR contributed to the conception and the design of the work. LR, PR, and IM were responsible for data collection, analysis, and interpretation of data. LR wrote the manuscript with valuable inputs from the remaining authors. RSC and IM made important intellectual contributions for the manuscript. All authors agreed for all aspects of the work and approved the version to be published.

## Conflict of Interest Statement

The authors declare that the research was conducted in the absence of any commercial or financial relationships that could be construed as a potential conflict of interest.
